# Effects of the Pentapeptide P33 on Memory and Synaptic Plasticity in APP/PS1 Transgenic Mice: A Novel Mechanism Presenting the Protein Fe65 as a Target

**DOI:** 10.3390/ijms20123050

**Published:** 2019-06-22

**Authors:** Titanilla Szögi, Ildikó Schuster, Emőke Borbély, Andrea Gyebrovszki, Zsolt Bozsó, János Gera, Róbert Rajkó, Miklós Sántha, Botond Penke, Lívia Fülöp

**Affiliations:** 1 Department of Medical Chemistry, Interdisciplinary Excellence Center, University of Szeged, Dóm tér 8, H-6720 Szeged, Hungary; szogititi@gmail.com (T.S.); schuster.ildiko@med.u-szeged.hu (I.S.); borbely.emoke@med.u-szeged.hu (E.B.); andrea.gyebrovszki@gmail.com (A.G.); bozso.zsolt@med.u-szeged.hu (Z.B.); gera.janos1990@gmail.com (J.G.); penke.botond@med.u-szeged.hu (B.P.); 2 Institute of Process Engineering, University of Szeged, Moszkvai Krt. 5-7, H-6725 Szeged, Hungary; rajko@mk.u-szeged.hu; 3 Institute of Biochemistry, Biological Research Centre, Temesvári Krt. 62, H-6726 Szeged, Hungary; santha.miklos@brc.mta.hu

**Keywords:** Alzheimer’s disease, amyloid beta peptide, amyloid beta precursor protein, Fe65 protein, WW domain, proline-rich peptide

## Abstract

Regulated intramembrane proteolysis (RIP) of the amyloid precursor protein (APP) leads to the formation of fragments, among which the intracellular domain of APP (AICD) was also identified to be a causative of early pathological events. AICD-counteracting proteins, such as Fe65, may serve as alternative therapeutic targets of Alzheimer’s disease (AD). The detection of elevated levels of Fe65 in the brains of both human patients and APP transgenic mice may further strengthen the hypothesis that influencing the interaction between Fe65 and APP may have a beneficial effect on the course of AD. Based on a PXP motif, proven to bind to the WW domain of Fe65, a new pentapeptide was designed and tested. The impedimental effect of P33 on the production of beta amyloid (Aβ) (soluble fraction and aggregated plaques) and on the typical features of the AD pathology (decreased dendritic spine density, synaptic markers, elevated inflammatory reactions) was also demonstrated. Significant enhancements of both learning ability and memory function were observed in a Morris water maze paradigm. The results led us to formulate the theory that P33 acts by altering the conformation of Fe65 via binding to its WW domain, consequently hindering any interactions between Fe65 and key members involved in APP processing.

## 1. Introduction

During the last decade, proteolytic products of the amyloid precursor protein (APP) different from beta amyloid (Aβ) became the targets of Alzheimer’s disease (AD) research, as Aβ could not be proven to possess a decisive role in the AD pathomechanism. The so-called regulated intramembrane proteolysis (RIP) [1] of APP requires the concerted action of proteases (secretases), as well as many intracellular proteins that bind to the intracellular domain of APP (AICD). AICD is formed both in the amyloidogenic and non-amyloidogenic pathways, and its interactome is wide and complex (e.g., the X11 protein family [2,3,4,5], Disabled-1 (Dab-1) [5,6], Shc-transforming protein [7,8], c-Jun N-terminal kinase-interacting proteins (JIP-1) [5], the Fe65 protein family [1,9,10], and Peptidyl-prolyl cis-trans isomerase NIMA-interacting- (Pin1 [9,11]). Interaction between AICD and these proteins can modulate Aβ levels, Tau phosphorylation, and transcriptional activity [9]; therefore, AICD is hypothesized to be strongly involved in the development of AD. The internalization of APP from the plasma membrane and intracellular trafficking regulate Aβ production [12].

**Figure 1:**
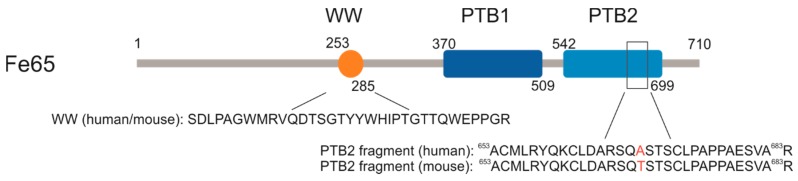
Human (Uniprot: O00213) and mouse (Uniprot: Q9QXJ1) domain structures of Fe65. WW (tryptophan, tryptophan) protein interaction domains are the same in human and mouse Fe65; PTB2 (phosphotyrosine-binding) domains differ only in one amino acid at position 668.

The Fe65 family (Fe65, Fe65L1, and Fe65L2) plays a principal role in APP processing and trafficking, as well as in actin cytoskeleton remodeling, cell motility, neuronal growth cone formation, synapse formation, synaptic plasticity, and consequently in the learning process [13,14,15]. Fe65 contains three domains: two C-terminal phosphotyrosine-binding (PTB1 and PTB2) regions and an N-terminal WW domain [13,16]; all of them are highly conserved. The mouse Fe65 WW domain is identical with that of human Fe65, while the PTB2 domains differ only in one amino acid in the two species (Figure 1).

Binding of Fe65 through its PTB2 domain to APP modulates the proteolytic processing and trafficking of the latter and, consequently, Aβ production as well [9,13,17]. However, literary data are contradictory whether Fe65 decreases or increases the level of Aβ, with results seeming to vary based on the applied experimental model [9,17].

In Fe65 transfected cells (human embryonic kidney cells 293 (HEK293), human H4 neuroglioma cells (H4), and Madin–Darby Canine Kidney cells (MDCK), overexpression of Fe65 increased the production of Aβ [18,19,20,21]. Coincidentally, silencing the Fe65-encoding gene (*APBB1*) caused reduced levels of Aβ secretion in an H4 cell culture [22]. Suh et al. [23] obtained identical results in a primary cortical neuron culture. Furthermore, in two Fe65/Fe65L1 knock-out (KO) mouse strains (Tg2576 and 129SvEv Bradley), the lack of Fe65 resulted in reduced Aβ levels [24,25].

In contrast, Ando and colleagues [11] found that Fe65 overexpression decreases the production of Aβ_1–40_ and Aβ_1–42_ in an HEK293 cell line transiently transfected with APP695, compared to the controls. Adjacently, it was found that a Glu^668^-APP mutation causes a significant increase in the levels of Aβ_1–40_ and Aβ_1–42_, presumably due to the decreased formation of pThr^668^-APP. This result was confirmed by experiments in double hFe65/APP (751 isoform carrying the Swedish and London mutation) and single APP transgenic mouse strains. Resulting from Fe65 overproduction, total Aβ and Aβ_1–42_ concentrations, as well as the number of Aβ plaques, were reduced in double transgenic mice in comparison with single APP transgenic animals [17].

The WW domain, which is also represented in Fe65, possesses a compact, antiparallel, three-stranded β-sheet structure providing an interface for binding linear peptide motifs [26,27]. WW domains are known to interact with special protein structures, the so-called proline-rich sequences [28,29]. The K_d_ of this binding typically falls in the low μM range [26,30]. This was also proven for the WW domain of Fe65, which anchors several proteins equipped with a common PXP motif [1,13,31,32,33], many of them having key roles in Fe65-mediated transcription mechanisms (SET [34]), kinase activation, actin polymerization (Mammalian-enabled protein (Mena [35])), and Aβ production (Glycogen synthase kinase 3 (GSK3β), c-Abl kinase [36]).

**Figure 2:**
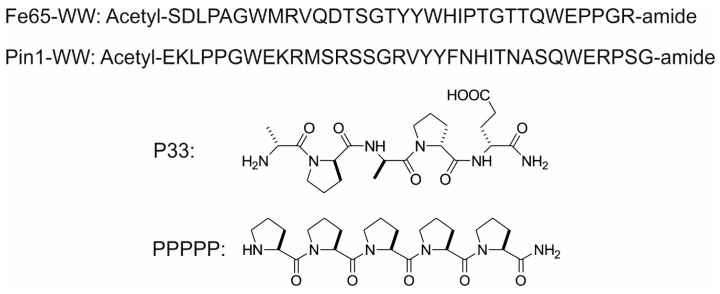
Sequences of the WW domains (Fe65-WW domain, Pin1-WW domain), and chemical structures of P33 and PPPPP.

As Fe65 activity bears great influence on APP processing and Aβ formation, the stereochemical regulation of Fe65 activity through its WW domain using proline-rich sequences might pioneer new therapeutic ways to suppress or modulate AD development. To achieve this goal, we sought to design a molecule with a specific PXP motif. The pentapeptide P33 (Figure 2), which is composed of d-amino acids, was examined in isothermal titration calorimetry (ITC) studies, and its affinity to bind to the WW domain of Fe65 (Figure 2) was determined. To verify the specificity of the stereochemical regulation of Fe65, we tested the binding of P33 to the WW domain of another protein, Pin1 (Figure 2), found to also participate in APP processing. While Pin1 has a neuroprotective effect in healthy neurons, under oxidative stress or in AD, decreased Pin1 levels shift APP processing toward the amyloidogenic pathway. Pastorino et al. [37] found decreased levels of Aβ in Pin1 overexpressing Chinese hamster ovarian (CHO) cells. Pin1 also modulates Tau phosphorylation [9,38]. Pin1 deficit causes Tau hyperphosphorylation [39], after which the hyperphosphorylated Tau aggregates into neurofibrillary tangles (NFTs) instead of undergoing microtubule polymerization.

In our complex in vivo study, being one of the widely used animal models of familial AD [40], APPswe/PS1ΔE9 double transgenic (APP/PS1) mice were treated together with C57BL/6 wild-type (WT) mice as controls for six months with P33. We tested the effect of the pentapeptide on spatial learning and on memory with the Morris water maze (MWM), and on dendritic spinal density using Golgi Cox impregnation. Synaptic function was further characterized by determining the amounts of postsynaptic density protein 95 (PSD95) and synaptophysin (SYN) with Western blots (WB). We also examined densities of the astrocytes (glial fibrillary acidic protein, GFAP), the microglia (ionized calcium-binding adapter molecule 1, Iba1), and the plaques (4G8) using immunohistochemical methods. The concentration of soluble Aβ was also determined with an enzyme-linked immunosorbent assay (ELISA). We also tested the effect of P33 on the Fe65, APP, pT^668^-APP, C83, and C99 levels of the mice. Our results suggest that elongated treatment with this pentapeptide may have beneficial effects on several biological processes (spatial learning, amyloid burden), presumably due to its influence on the Fe65 pathway.

## 2. Results

### 2.1. P33 Binds to Fe65-WW but Not to Pin1-WW In Vitro

**Figure 3:**
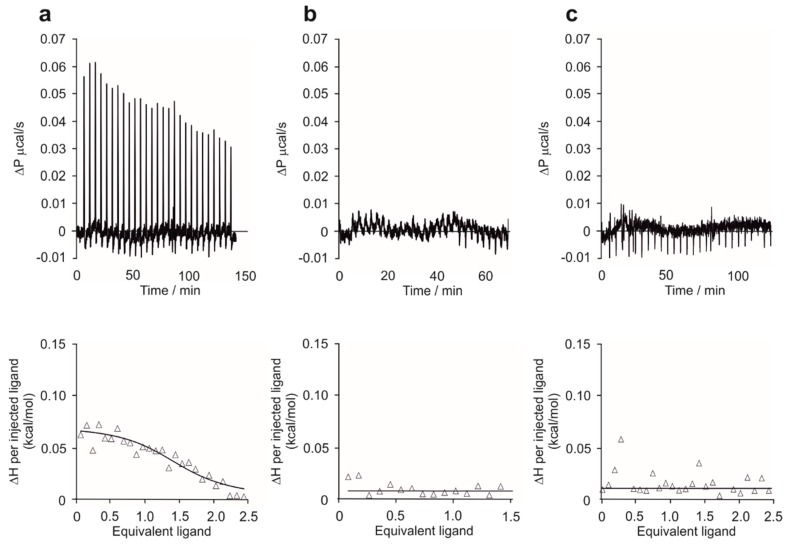
Isothermal titration calorimetry (ITC) measurements. Raw ITC data (top) and the integrated heat data relative to the molar ratio (bottom) in the interaction of P33 and Fe65-WW (**a**), PPPPP and Fe65 (**b**), and P33 and Pin1 (**c**) in phosphate-buffered saline (PBS) (pH 7.4). (**a**) N = 1.14 ± 0.01, ΔH = 0.082 ± 0.001 kcal∙mol^−1^, K_d_ = 4.68 ± 0.04 μM; (**b**) no binding observed; (**c**) no binding observed.

Binding studies were conducted applying ITC measurements. The thermodynamical parameters of the binding interactions between P33 and the WW domain of Fe65 and Pin1 were determined in order to reveal the difference in the two WW domains in question regarding their binding with the pentapeptide. The titration of the Fe65-WW domain with P33 at 310 K resulted in the dissociation constant K_d_ = 4.68 ± 0.04 μM, calculated from the binding isotherm (Figure 3a). The stoichiometric factor N = 1.14 indicates an equimolar interaction and, thus, the formation of a [P33]/[Fe65-WW] complex in a 1:1 ratio.

In a control experiment with PPPPP, no binding between the Fe65-WW and the pentapeptide could be observed at 310 K (Figure 3b), thereby proving that the unique structure of P33 is necessary for the binding interaction. 

On the other hand, the titration of Pin1-WW with P33 at 310 K showed no considerable binding (Figure 3c) either. These results suggest that P33 can bind selectively to the Fe65-WW domain, which verifies the decisive role of a possible P33/Fe65-WW interaction in APP processing, instead of a Pin1-WW-involved mechanism.

### 2.2. P33 Treatment Does Not Influence Key Protein Levels Involved in the Fe65/APP Route in the Transgenic Mice

**Figure 4:**
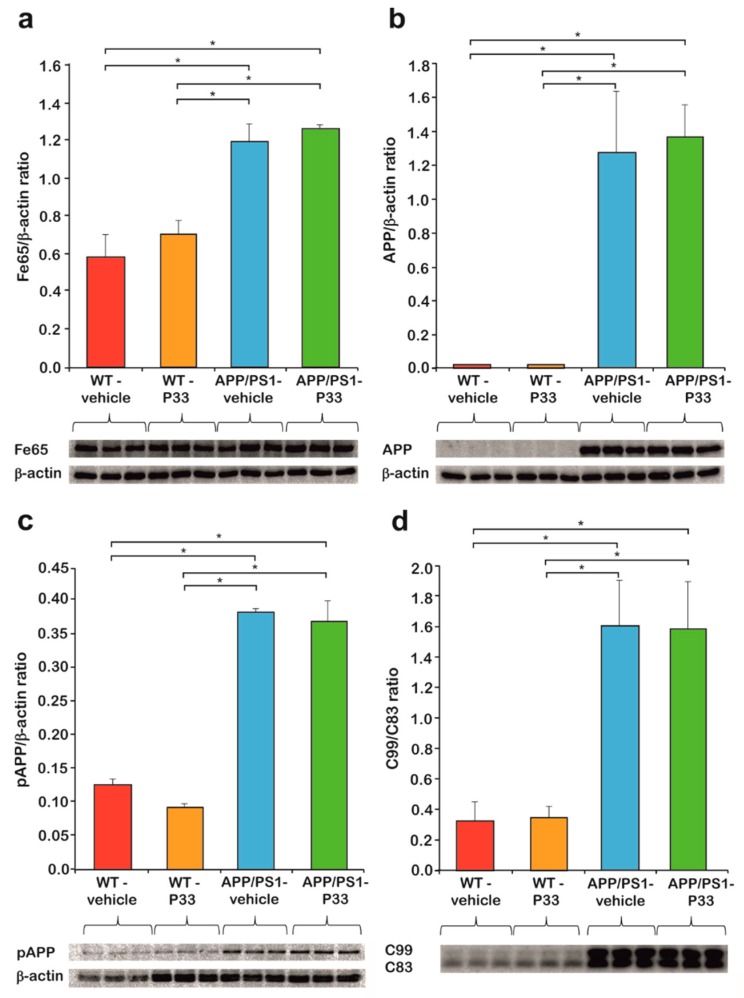
Western blot (WB) analysis of Fe65, amyloid precursor protein (APP), pThr^668^-APP, C83, and C99 levels. (**a**) Fe65 level of the APP/PS1 mice is significantly higher than in the wild-type (WT) animals, which did not change upon P33-treatment (* represents significant differences at the following *p*-levels: H_3,8_ = 5.702, *p* = 0.022; APP/PS1-vehicle vs. WT-vehicle, WT-P33 *p* = 0.001, 0.003; APP/PS1-P33 vs. WT-vehicle, WT-P33 *p* ≤ 0.0001, *p* = 0.002; WT-vehicle vs. WT-P33 *p* = 0.625; APP/PS1-vehicle vs. APP/PS1-P33 *p* = 0.517). (**b**) Human APP was observed only in transgenic mice, the level of which did not change with P33-treatment either (* represents significant differences at the following *p*-levels: APP/PS1-vehicle vs. APP/PS1-P33 *p* = 0.622). (**c**) pThr^668^-APP level of APP/PS1 mice is significantly higher compared to the WT animals. The P33-treatment did not alter the level of pThr^668^-APP (* represents significant differences at the following p-levels: H_3,8_ = 98.096, *p* ≤ 0.0001; APP/PS1-vehicle vs. WT-vehicle, WT-P33 *p* ≤ 0.0001, 0.0001; APP/PS1-P33 vs. WT-vehicle, WT-P33 *p* ≤ 0.0001, 0.0001; WT-vehicle vs. WT-P33 *p* = 0.183; APP/PS1-vehicle vs. APP/PS1-P33 *p* = 0.554). (**d**) C99/C83 ratio of APP/PS1 transgenic mice is higher than in the WT animals, which was not affected during the P33-treatment (* represents significant differences at the following *p*-levels: H_3,8_ = 32.344, *p* ≤ 0.0001; APP/PS1-vehicle vs. WT-vehicle, WT-P33 *p* ≤ 0.0001, 0.0001; APP/PS1-P33 vs. WT-vehicle, WT-P33 *p* ≤ 0.0001, 0.0001; WT-vehicle vs. WT-P33 *p* = 0.807; APP/PS1-vehicle vs. APP/PS1-P33 *p* = 0.926).

Three-month-old APP/PS1 double transgenic and WT mice were injected for six months with P33. To determine the effect of P33 administration on the Fe65, APP, pT^668^APP, C83, and C99 levels, hippocampus (HC) and cerebral cortex (CTX) regions of the brains of nine-month-old animals were subjected to WB analyses. We found significantly elevated endogenous Fe65 concentrations in transgenic mice compared to the WT animals (H_3,8_ = 5.702, *p* = 0.022; APP/PS1-vehicle vs. WT-vehicle, WT-P33 *p* = 0.001, 0.003; APP/PS1-P33 vs. WT-vehicle, WT-P33 *p* ≤ 0.0001, *p* = 0.002; WT-vehicle vs. WT-P33 *p* = 0.625; APP/PS1-vehicle vs. APP/PS1-P33 *p* = 0.517), which could not be considerably changed by the treatment with P33 (Figure 4a).

Furthermore, 6E10 recognizes only human but not mouse APP, which could also be proven by our WB studies, as no detectable amounts of APP were present in WT mice. The amount of APP in the APP/PS1 animals did not change with the P33-treatment (APP/PS1-vehicle vs. APP/PS1-P33 *p* = 0.622, Figure 4b).

APP has eight phosphorylation positions in the cytoplasmic region, among which phosphorylation at T^668^ is held responsible for the binding of APP to Fe65, and for the consequent nuclear translocation of APP, playing a key role in the APP metabolism [18,41]. In accordance with the literature data [41], the phosphorylation at T^668^ was found to be significantly higher in the transgenic animals than in the WT ones, while the P33-treatment had no significant effect on the pT^668^-APP level (H_3,8_ = 98.096, *p* ≤ 0.0001; APP/PS1-vehicle vs. WT-vehicle, WT-P33 *p* ≤ 0.0001, 0.0001; APP/PS1-P33 vs. WT-vehicle, WT-P33 *p* ≤ 0.0001, 0.0001; WT-vehicle vs. WT-P33 *p* = 0.183; APP/PS1-vehicle vs. APP/PS1-P33 *p* = 0.554, Figure 4c). 

C83 is produced during the non-amyloidogenic processing of APP, while C99 is formed during the amyloidogenic pathway. The C99/C83 ratio is hypothesized, therefore, to provide information about the balance between the two pathways. As the amyloidogenic processing is increased in the transgenic mice, an elevated C99/C83 ratio could be observed, which was not influenced by the P33 treatment considerably (H_3,8_ = 32.344, *p* ≤ 0.0001; APP/PS1-vehicle vs. WT-vehicle, WT-P33 *p* ≤ 0.0001, 0.0001; APP/PS1-P33 vs. WT-vehicle, WT-P33 *p* ≤ 0.0001, 0.0001; WT-vehicle vs. WT-P33 *p* = 0.807; APP/PS1-vehicle vs. APP/PS1-P33 *p* = 0.926, Figure 4d).

### 2.3. P33 Restores the Pathologically Reduced Spine Density and Protects the Synapses

**Figure 5:**
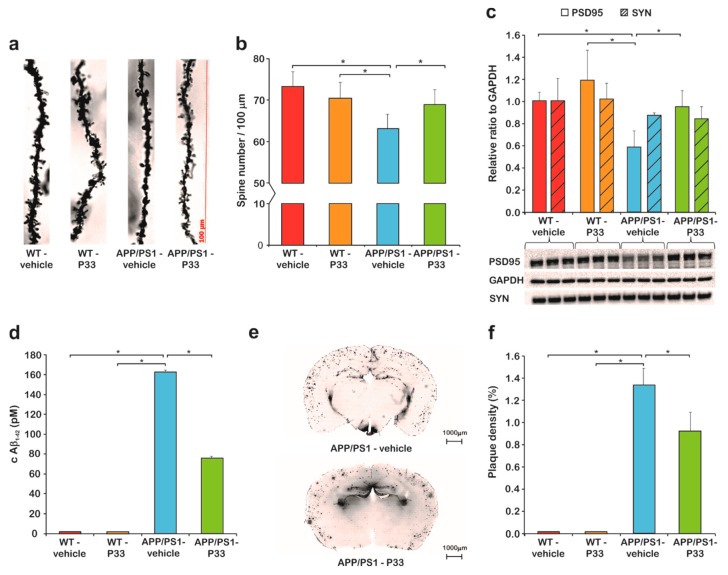
Histology results I. (**a**) The apical dendritic spine density in the CA1 region of the hippocampus (HC) was monitored. Representative photomicrographs of oblique dendritic segments stemming from WT-vehicle, WT-P33, APP/PS1-vehicle, and APP/PS1-P33 mice (100× magnification; scale bar: 100 μm). (**b**) Golgi staining revealed changes in the spine density after P33-treatment. Quantitative analysis of the apical dendritic spine density proved that P33 protected the synaptic terminals (*p* = 0.015). An impaired density was measured in APP/PS1-vehicle mice compared to WT-vehicle (*p* = 0.002), WT-P33 (*p* = 0.019), and APP/PS1-P33 (*p* = 0.041) animals. Bars represent the mean + standard error of the mean (SEM). * represents significant differences at the corresponding *p*-levels. (**c**) A significantly decreased PSD95 level was observed in APP/PS1-vehicle cohort compared to WT-vehicle, WT-P33, and APP/PS1-P33 animals (* represents significant differences at the following *p*-levels: H_3,8_ = 5.473, *p* = 0.024, APP/PS1-vehicle vs. WT-vehicle, WT-P33, APP/PS1-P33 *p* = 0.026, 0.004, 0.036). P33-treatment influenced the postsynaptic density protein 95 (PSD95) level considerably, and the statistical analysis revealed that the chronic compound treatment resulted in a significantly higher PSD95 level in APP/PS1-P33 mice compared to the APP/PS1-vehicle ones. Regarding synaptophysin (SYN) level, there is no difference between the four experimental groups (H_3,8_ = 1.573, *p* = 0.270) (**d**) Concentration of soluble amyloid beta (Aβ) in cerebral cortex (CTX) and HC: Aβ_1–42_ level of the vehicle or P33-treated WT and APP/PS1 mice measured by ELISA. No human Aβ_1–42_ was detected in the WT animals. The P33-treatment reduced the Aβ_1–42_ level in the transgenic mice (*p* < 0.0001). * represents significant differences at the corresponding *p*-levels. (**e**) Representative pictures of the 4G8 staining of amyloid plaques. (**f**) Measurements of Aβ plaque density. Aβ plaques could not be detected in WT animals. The P33-treatment influenced the plaque density considerably; the quantitative analysis revealed that chronic P33-treatment resulted in a significant decrease in the density of Aβ deposits in the APP/PS1-P33 group compared to that of the APP/PS1-vehicle animals (*p* = 0.013). * represents significant differences at the corresponding *p*-levels.

Golgi staining was used to assess the hippocampal apical dendritic spine density of CA1 pyramidal neurons after P33 or vehicle treatment, in WT and APP/PS1 animals. During this procedure, all types of spine were analyzed. At nine months of age, there was a significant difference between the groups (one-way ANOVA; F_3,18_ = 4.732, *p* = 0.015). A significant reduction in the spine density could be detected in the APP/PS1-vehicle group (Figure 5a,b), compared to WT-vehicle (*p* = 0.002), and WT-P33 (*p* = 0.019) animals, while treating the APP/PS1 mice with P33 was able to normalize the diminished spine density (APP/PS1-vehicle vs. APP/PS1-P33 *p* = 0.041).

To further explore the mechanism of P33 on synapses in our AD animal model, we examined the expression of synapse associated proteins PSD95 and SYN. In SYN level, significant differences were not found between the groups (Figure 5c, H_3,8_ = 1.573, *p* = 0.270). The expression of PSD95 was significantly decreased in APP/PS1 vehicle-treated mice compared to the other three, which could be restored by applying P33 on APP/PS1 mice, which demonstrates the positive effect of the P33 on synapse groups (Figure 5c, H_3,8_ = 5.473, *p* = 0.024, APP/PS1-vehicle vs. WT-vehicle, WT-P33, APP/PS1-P33 *p* = 0.026, 0.004, 0.036, respectively).

### 2.4. P33 Significantly Reduces the Amounts of Both Soluble and Deposited Aβ Forms in the APP/PS1 Animals

The amount of soluble Aβ_1–42_ measured along with the plaque density provides information on the development of AD in the applied animal model. The soluble fraction of Aβ_1–42_ was isolated from the HC and CTX regions of the brains of nine-month-old animals, by following the method of Shankar et al. [42], and quantitatively measured with a commercially available ELISA test, which selectively recognizes human Aβ_1–42_ [43] and is proven to show no cross-reaction with the full-length APP. The WT mice had no detectable amounts of Aβ_1–42_ (0 pM), proving that the 3D6 antibody supplied with the kit detects only human, not mouse Aβ. In the APP/PS1-vehicle group, a significantly higher Aβ_1–42_ concentration could be observed than in the APP/PS1-P33 group (Figure 5d, 162.70 pM and 75.86 pM, respectively, *p* < 0.0001). The results indicate that the treatment with P33 led to a decreased level of Aβ_1–42_ in the APP/PS1 mice. 

In order to examine the Aβ plaque load of both hippocampal and cortical areas, the density (%) of Aβ plaques was measured (Figure 5e,f). One-way ANOVA revealed a significant difference between the groups (F_3,16_ = 48.575, *p* ≤ 0.001). Brains of WT animals were completely devoid of any Aβ plaque depositions. The post hoc analysis proved that long-term administration of the compound had significantly decreased the plaque density in APP/PS1-P33 mice compared to the APP/PS1-vehicle (*p* = 0.013).

### 2.5. P33 Hinders Inflammatory Processes in the Mouse Brain

**Figure 6:**
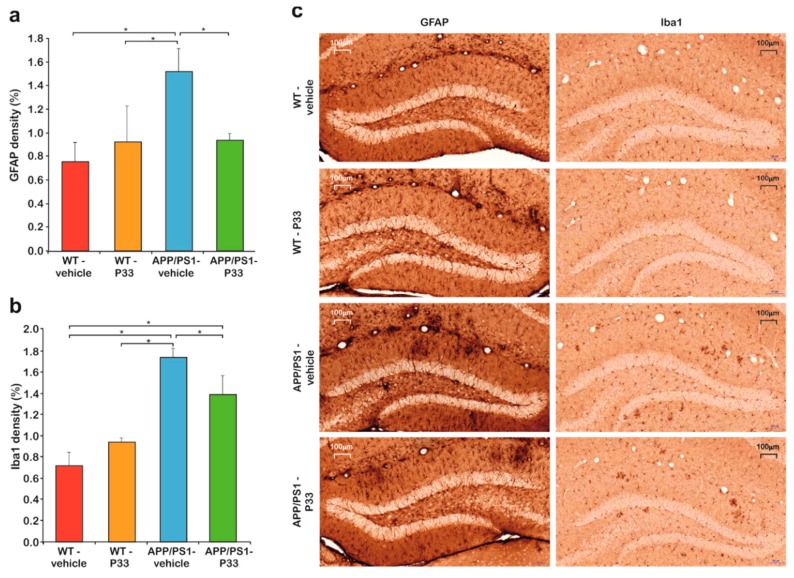
Histology results II. (**a**) Quantitative results of the glial fibrillary acidic protein (GFAP) staining. Significant differences were observed between the groups in the densities of hyperreactive astrocytes (*p* = 0.048). An increased GFAP-positive cell density was detected in the APP/PS1-vehicle group compared to the WT-vehicle (*p* = 0.009), the WT-P33 (*p* = 0.047), and the APP/PS1-P33 (*p* = 0.039) ones. * represents significant differences at the corresponding *p*-levels. (**b**) Results of the ionized calcium-binding adapter molecule 1 (Iba1) immunostaining. A significantly higher level of the activated microglia density (*p* = 0.004) was found in the APP/PS1-vehicle animals than in the other groups (WT-vehicle: *p* = 0.001, WT-P33: *p* = 0.003, APP/PS1-P33: *p* = 0.046). * represents significant differences at the corresponding p-levels. (**c**) Representative images of GFAP and Iba1 stainings. Scale bars represent 100 μm.

Neuroinflammation is considered to be the result of the activation of the innate immune system in central nervous system (CNS) with a protective function against infections, injuries, and neurodegenerative processes. The inflammatory response is mediated by activated microglia and hyperreactive astrocytes. To examine the effect of long-term P33 administration on these cell types, immunohistochemical stainings were performed. GFAP staining revealed that the density of hyperreactive astrocytes was higher in the whole HC and CTX of the APP/PS1-vehicle group, than in the other three groups (Figure 6a, one-way ANOVA F_3,16_ = 3.343, *p* = 0.048, Fisher’s least significant difference (LSD) post hoc tests: APP/PS1-vehicle vs. WT-vehicle, WT-P33, APP/PS1-P33 *p* = 0.009, 0.047, 0.039, respectively). Microglial density was examined by Iba1 immunostaining in HC and CTX areas. The density of Iba1-positive microglia was higher in the APP/PS1-vehicle treated mice than in the other groups (Figure 5b,c, one-way ANOVA F_3,16_ = 6.629, *p* = 0.004, Fisher’s LSD post hoc tests: APP/PS1-vehicle vs. WT-vehicle, WT-P33, APP/PS1-P33 *p* = 0.001, 0.003, 0.046, respectively).

### 2.6. P33 Exerts a Positive Effect on the Learning Ability and Memory Functions in an MWM Paradigm

**Figure 7:**
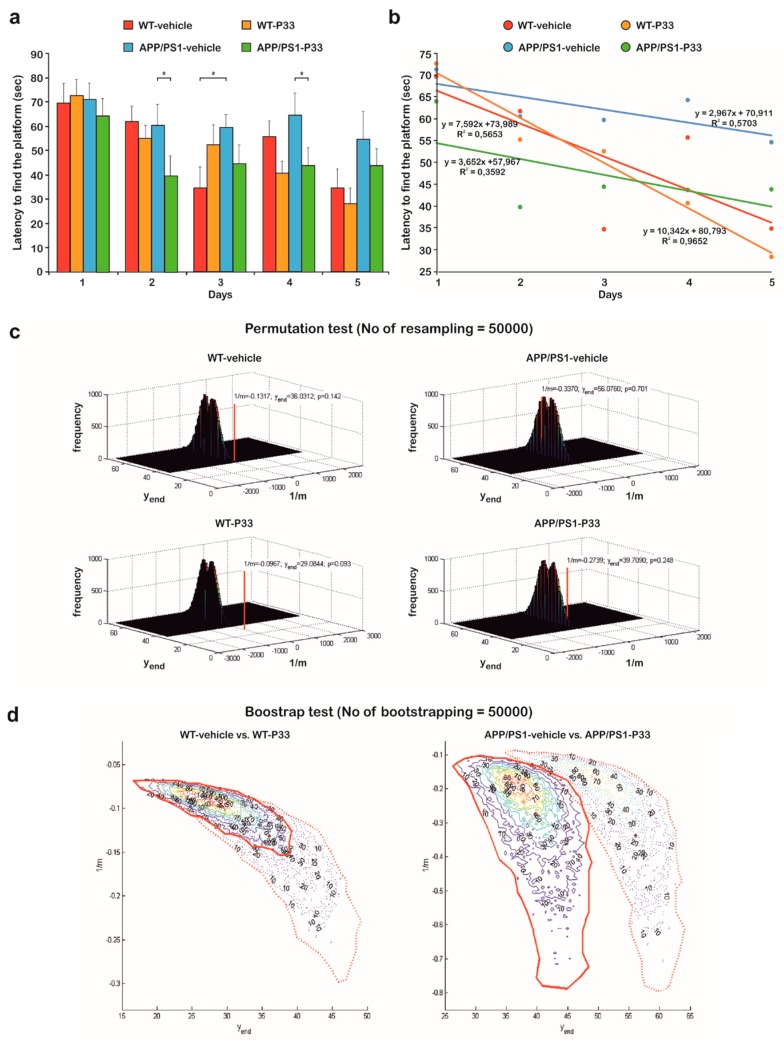
Statistical analysis of the behavior studies. (**a**) The uncertainty and the trend of every latency data versus days on a bar chart. (* represents significant differences at the following *p*-levels: F_3,181_ = 3.365, *p* = 0.030; APP/PS1 vs WT-vehicle, WT-P33, APP/PS1-P33, *p* = 0.033, 0.015, 0.006, respectively) (**b**) Former latency data to find the platform in seconds against days. For each case, trend lines were fitted onto the average swimming times. The fitted equations and the determination coefficient (*R^2^*) values are also shown. (**c**) Three-dimensional (3D) distribution results of the permutation tests. WT mice results are significantly different from randomness (positions of the red lines fall far from the distribution of the randomly generated data), while the APP/PS1-vehicle treated mice measurements can be considered as a random event generation (positions of the red line fall in the distribution of the randomly generated data). P33-treatment led to a significant improvement, as the randomness of the measurements was drastically decreased. (**d**) Two-dimensional (2D) contour diagrams of wild-type and transgenic group data distributions based on bootstrapping. The left panel depicts that the WT groups have a high intersection area (32% and 89% of the total areas in case of vehicle and P33-treatments, respectively), indicating that there is no significant difference in the two descriptive parameters between the groups; both were able to learn the task similarly. On the right panel, in case of the APP/PS1 groups, the intersection area is small (approximately 2% of the total areas of both vehicle and P33-treatments), which indicates a significant difference in the parameters between the groups, emphasizing the difference in the learning abilities of the P33-treated vs. non-treated groups.

To elucidate the effects of P33 on spatial learning and memory, an MWM test was conducted for five days following the administration. Upon evaluation, we performed a mixed ANOVA in which significant differences were found between the parameters (mixed ANOVA, F_3,181_ = 3.365, *p* = 0.030). The post hoc analysis of the results yielded that the APP/PS1-vehicle group had a significantly bigger latency to find the platform in comparison with the other groups (WT-vehicle, *p* = 0.033; WT-P33, *p* = 0.015; APP/PS1-P33, *p* = 0.006), which confirms a learning and memory deficit in untreated transgenic mice, whereas P33 seemed to have a positive effect on their learning abilities (Figure 7a,b).

Since the measured times did not have a Gaussian distribution and because of the problems in the correct interpretation and/or determination of the probabilities of the false positive and negative decisions in cases of multiple testing, an investigation was needed to determine the differences between the groups. Figure 7a depicts the average swimming times on a bar plot. For each case, trend lines were fitted onto the average swimming times, and *R^2^* values of the fitted points were also calculated (Figure 7b). For the correct determination of the probability of the false positive decision error, a permutation test was applied, in which 50,000 resamples were generated by independently mixing all data related to both the mice and the days. The reciprocals of the slopes (1/m) of the fitted straight lines between the swimming times and the days, and swimming time data of the last days (y_end_) were calculated and plotted onto three-dimensional (3D) distribution maps. The empirical resampled probabilities were generated for all four measured datasets (two different mice with two different treatments; calculated using the average swimming times for each) in the two-dimensional (2D) data space for getting the estimations of the probabilities of the false positive decision error; farther from the bulk of the resampled values of two variables (and closer to the origin) means a more pronounced difference from the randomness (Figure 7c). It can be concluded that WT mice results are significantly different from randomness (position of the red lines fall far from distribution of the randomly generated data), while the APP/PS1-vehicle treated mice measurements can be practically considered as a random event generation (position of the red line falls in the distribution of the randomly generated data). P33-treatment led to a significant improvement, as the randomness of the measurements was drastically decreased. 

Secondly, bootstrap resampling with 50,000 iteration steps was used to generate a new dataset independently from that used for the permutation test to reveal the differences between the swimming times of the wild-type and transgenic groups. Again, the reciprocal of the slopes of the fitted straight lines (1/m) and swimming time data of the last day (y_end_) were calculated and plotted onto 2D contour maps in this case. Red diamond and red dotted contour lines represent the vehicle treatments, while red circle and red continuous contour lines show the P33 treatments. The bolded continuous and dotted lines depict 90% of the generated data. Thus, the conclusion can be drawn with a probability level of 0.9. In Figure 7d (left panel), the WT groups have a high intersection area (32% and 89% of the total areas in case of vehicle and P33-treatments, respectively), which indicates that there is no significant difference in the two descriptive parameters between the groups; both were able to learn the task similarly. In the case of the APP/PS1 groups (Figure 7d, right panel), the intersection area is small (approximately 2% of the total areas of both vehicle and P33-treatments), which indicates a significant difference in the parameters between the groups, emphasizing the difference in the learning abilities of the P33-treated vs. non-treated groups. Furthermore, this visualization method demonstrates that our measurement data cannot be evaluated with classical parametric and even non-parametric test-methods, because the distributions are non-symmetrical, as the contours are not elliptical. In general, if the measured data are influenced by a systematic effect due to the learning procedure, the assumptions for the ordinary test-methods may be not fulfilled; thus, resampling methods are recommended.

## 3. Discussion

The latest results in AD research concerned the role of key players different from Aβ or Tau in the pathomechanism of the disease. RIP of APP involves many putative proteinaceous targets to be studied and subjected to drug design. We aimed to find new sequences based on the common PXP motif, which might be able to modify the action of the WW domain-bearing Fe65, whereby an advantageous effect on the disease pathology could be achieved. Our model of choice was the APP/PS1 transgenic mouse, which, as we tested, overexpresses its own endogenous Fe65 (mFe65), presumably as a result of a feedback effect of the human APP (hAPP) overexpression. This result is not surprising and it also bears an analogy with human AD symptoms. In another hAPP-overexpressing mouse model [44], hAPP overexpression was shown to produce elevated mFe65 levels [45], while post mortem analysis of human sporadic AD brain samples revealed that, in the hippocampal areas, an increase in Fe65 immunoreactivity was associated with the severity of the disease [46]. These findings strengthen our hypothesis that the pathogenic overexpression of Fe65 might serve as a starting point to find new therapeutic ways for curing AD, and that the APP/PS1 transgenic model can be applied to study molecules for that purpose.

Targeting WW domains represented in several proteins involved in AD raises the question of specificity. To address this, we searched for sequences of protein molecules in protein databases which bear WW domains and are also involved in AD pathology. Besides Fe65, we identified another one, Pin1, which participates in the Tau pathology, as is able to restore the microtubule binding affinity of Tau [27] by binding to its phospho-T^668^P motif. Our newly designed pentapeptide, P33, was hypothesized to interact with the WW domains of both Fe65 and Pin1, thereby directly influencing the pathomechanism of AD through both Aβ and Tau pathologies.

Chemical synthesis of the discrete WW domains of the relevant proteins enabled the application of in vitro methods, via which the binding affinity of P33 could be determined. Our ITC measurements confirmed the binding of P33 to the Fe65-WW with a low micromolar K_d_, which falls in the range generally observed in similar experiments. Interactions between certain WW domains and proline-rich sequences possessed dissociation constants ranging from high nM to low mM values [47,48]. On the other hand, no detectable binding to the Pin1-WW could be observed.

The results verify the selective binding of P33 to Fe65 and, therefore, we can assume that the mechanism is related to APP processing, but the Tau pathology remains unaffected. The selectivity of the binding can be explained with the differences in the respective structures of the WW domains; according to a generally accepted classification, Fe65-WW belongs to Group II, the members of which recognize the PPLP motif preferentially, while Pin1-WW is in Group IV, and is, therefore, supposed to interact mostly with a (phospho-S/phospho-T)P unit of a proline-rich sequence [49].

Our results unequivocally confirmed the advantageous effect of the P33-treatment on the memory, learning ability, and inflammatory processes in the applied transgenic animal model. Moreover, we could detect decreased amounts of both soluble and aggregated Aβ in the P33-treated transgenic animals. Soluble Aβ aggregates are considered to be trigger signals to induce dendritic spine loss and synapse dysfunction at the early stage of AD, correlating with learning impairments and memory deficits [50,51]. Recently, the synaptic protection and repair became important in AD research as key processes to ameliorate cognitive functions. In our experiments, the positive effects of P33 were demonstrated on dendritic spines and postsynaptic proteins (PSD95), together with the spatial learning and memory performances. 

In order to explain the observed beneficial effects, we attempted to identify the key step in the mechanism, in which P33 should influence the action of Fe65 in the downstream processes. A series of Western blot studies proved that neither an alteration in the APP expression nor its pThr^668^-induced binding to Fe65, and a hypothesized shift in the ratio of the amyloidogenic/non-amyloidogenic processing pathways could not be held responsible for the effects exerted by P33. Therefore, we put forth that P33 acts simply by binding to the WW domain of the Fe65, consequently hindering its further interaction with other members of the pathophysiological processes. According to literature data, this “blockade” might take effect through an alteration of the bioactive conformation of Fe65. Cao et al. [52] proposed that Fe65 possesses an inactive closed conformation, in which the WW domain binds to the PTB2 domain. APP and a certain membrane-associated factor together are able to open the inactive form of Fe65, whereby the latter can be activated. Feilen et al. [53] showed that Fe65 not only adopts a closed conformation, but that, in the absence of AICD, it also tends to form a homodimer through the APP-binding site. They also found that the PTB2 domain is not able to bind its own WW domain and the AICD simultaneously, since its interaction with the AICD weakens the binding to the WW, resulting in a partial opening of the inactive form [52,53]. APP and other membrane-associated factors (e.g., PIP2) together at the cell membrane induce the dissociation of the homodimer and the opening of Fe65 simultaneously, with the formation of an Fe65–APP complex [53]. 

However, the influence of this activation mechanism on AD pathology is still debated in the literature. To our best knowledge, the observed biological effects of Fe65 could mainly be explained by the altered expression of the protein, not by the structural changes in the bioactive conformation; however, the results are highly contradictory. Accordingly, in some animal models, the absence of Fe65 (Fe65 KO mice [24]) or in other cases the overexpression of Fe65 (Fe65/APP mice [9,17]) resulted in decreased amyloid levels. In our paradigm, the Fe65 levels of the mice were proven to be identical in the P33- and the vehicle-treated transgenic animal groups; therefore, we hypothesize that the observed significant decrease in amyloid level is due to changes in the Fe65 conformation, initiated by the interaction of P33 with the WW domain of the protein. The resulting P33–Fe65 complex may decrease the quantity of the Fe65/APP complex considerably, the formation of which was proven to be an important step in APP processing, consequently leading to decreased Aβ production.

In a comprehensive review, Bórquez et al. [1] summarized the so-called Fe65-interactome and also listed the binding sites, as well as the functions, of the interacting proteins. Among these, the WW-interacting ones were also proven to contain PXP motifs, through which they are attached to Fe56. Therefore, we cannot exclude the possibility that such protein–protein interactions that may have a consequent effect on the Aβ production might be modulated by P33 as well. Mena, Abl kinase, and GSK-3β are such candidates, and their roles in AD are widely studied. However, starting from the represented results, further research is needed to get a deeper insight into the mechanisms through which compounds like P33 might influence the effect of these proteins in the course of AD.

## 4. Materials and Methods 

### 4.1. Materials

N-terminally protected amino acids were purchased from Orpegen (Heidelberg, Germany), Bachem (Bubendorf, Switzerland), and GL Biochem (Shanghai, China). *N*,*N*′-Dicyclohexyl-carbodiimide (DCC), 1-hydroxybenzotriazole (HOBt), 1 [bis(dimethylamino)-methylene]-1*H*-1,2,3-triazolo[4,5-b]pyridinium-3-oxid hexafluorophosphate (HATU), and 4-methylbenzhydrylamine hydrochloride (MBHA × HCl) resin were purchased from GL Biochem (Shanghai, China), and Rink Amide resin was obtained from Bachem (Bubendorf, Switzerland). Solvents and *N*,*N* diisopropylethylamine (DIPEA) were obtained from Sigma-Aldrich (St. Louis, MO, USA). HPLC-grade trifluoroacetic acid (TFA) was ordered from Pierce (Rockford, IL, USA). 

### 4.2. Synthesis

For the synthesis of P33 (for structure see Figure 2), standard Boc chemistry was used on an MBHA × HCl (1.9 mmol∙g^−1^) resin, with DCC/HOBt activation. The peptide was cleaved from the resin with a cleavage cocktail containing 10 mL of HF, 0.8 mL of dimethyl-sulfide, and 0.2 mL of anisole for 1 g of peptide-resin, at 0 °C, for 45 min. The crude peptide was precipitated with diethyl-ether, dissolved in 50% acetonitrile (ACN)/H_2_O and lyophilized.

Synthesis of acetyl-Fe65-WW, acetyl-Pin1-WW, and the control PPPPP (structures involved in Figure 2) was carried out on a Rink Amide AM (0.3 mmol∙g^−1^) resin using Fmoc chemistry and activation with HATU in the presence of DIPEA. Cleavage was achieved by a treatment with TFA/H_2_O/triisopropylsilane/1,4-dithio-dl-threitol/phenol (90:5:3:1:1) at room temperature (RT). TFA was removed in vacuo, the peptides were then precipitated using cold, dry diethyl-ether, dissolved in ACN/H_2_O and lyophilized.

### 4.3. Purification

Peptides were analyzed and purified using RP-HPLC. 0.1% TFA in deionized (d.i.) water and 80% ACN, 0.1% TFA in d.i. water were used as eluent A and eluent B, respectively. 

Analytical HPLC experiments were performed on a Hewlett-Packard Agilent 1100 Series HPLC apparatus using a Luna C18 column (100 Å, 5 μm, 250 × 4.60 mm, Phenomenex, Aschaffenburg, Germany), at a flow rate of 1.2 mL∙min^−1^ with a gradient of 0 to 25% B over 25 min.

Preparative chromatography was carried out on a Shimadzu HPLC instrument equipped with a Luna C18 column (100 Å, 10 μm, 250 × 21.2 mm, Phenomenex, Aschaffenburg, Germany) at a flow rate of 4 mL∙min^−1^. The gradient was 0 to 30% B over 60 min for P33, 30% to 60% B over 100 min for Fe65-WW, 20% to 60% B over 120 min for Pin1-WW, and 0 to 40% B over 80 min for PPPPP.

### 4.4. ITC

Isothermal titrations were performed using a Microcal VP-ITC (Malvern Instruments, Malvern, UK) microcalorimeter. Binding experiments were carried out in degassed phosphate-buffered saline (PBS) at pH 7.4. The concentrations of Fe65-WW and Pin1-WW were normalized for the peptide contents. In one titration experiment, 10-μL portions of the ligand-containing solution (P33 or PPPPP) were injected from a computer-controlled 300-μL microsyringe at intervals of 300 s into the solution of the WW domain (Fe65-WW or Pin1-WW), dissolved in the same buffer as for the ligand. The rev of the microsyringe was set to 307 rpm. Measurements were repeated twice at 310 K. The starting concentrations of Fe65-WW and Pin1-WW in the cell were 83.5 and 81.4 μM, respectively, and the concentrations of the stock solutions containing P33 or PPPPP in the syringe were 1.006 and 1.056 mM, respectively. Control experiments were performed by injecting P33 or PPPPP into the cell containing plain buffer without the WW domain, with results achieved via subtracting the dilution heats from those measured in the presence of the WW domain. 

### 4.5. Animals

For this study, three-month-old male and female WT and APP/PS1 mice (*n* = 37) were used. Four groups were defined: WT-vehicle (physiological saline) (male *n* = 4; female *n* = 6), WT-P33 (male *n* = 6; female *n* = 5), APP/PS1-vehicle (male *n* = 3; female *n* = 5), and APP/PS1-P33 (male *n* = 6; female *n* = 2). Animals were subjected to intraperitoneal (i.p.) injections of P33 (5 mg∙kg^−1^) for five days per week, over a course of six months. The mice were kept in groups under constant temperature (23 ± 0.5 °C), lighting (12-h/12-h light/dark cycle, lights on at 7:00 a.m.), and humidity (~50%). Standard mouse chow and tap water were supplied ad libitum. All behavioral experiments were performed in the light period. Handling was done daily at the same time. 

Experiments were performed in accordance with the European Communities Council Directive of 22 September 2010 (2010/63/EU on the protection of animals used for scientific purposes) and were approved by the regional Station for Animal Health and Food Control (Csongrád-county, Hungary; project identification code: XVI/1248/2017, 20th March 2017). Formal approval to conduct the experiments was obtained from the Animal Experimentation Committee of the University of Szeged (project identification code: XXVI./3644/2017, 29th November 2017).

### 4.6. MWM

Spatial learning and memory were analyzed in MWM. A circular pool (d = 130, h = 60 cm), filled with water (23 ± 1 °C), served as the maze bound by a black curtain with some high-contrasted distal paper cues. Additionally, some other spatial objects were visible from the pool (e.g., lamps, video camera). The water was opalized with milk. The maze was segmented into four virtual quadrants with the platform (d = 10 cm) positioned in the middle of one of these (target quadrant), set 0.5 cm below the water surface. The room was illuminated by three lamps diffusely lighting all points of the maze with approximately equal intensity.

The experiment lasted for five days, and was conducted on every occasion in the light period. Mice were tested twice every day when they were allowed to swim until they found the platform. If the animal failed to find the platform within 90 s, it was guided to it or placed manually on top of it for 15 s. Four different starting points were used during the experiment; starting points were changed randomly during the tests. Trajectories of the mice were recorded with a video camera fixed on the ceiling, equipped with the software EthoVision XT8 (Noldus Information Technology, The Netherlands, 2011). The tracking system calculated the time to reach the platform, swimming speed, length of the swimming path (distance), and percentage time spent in each of the four virtual quadrants during the trials.

### 4.7. ELISA

Nine-month-old mice (three animals per group) were euthanized via cervical dislocation. Their brains were removed, and the tissue containing the HC and the CTX was quickly dissected and stored at −80 °C until further use. The tissue was homogenized on ice, in a buffer containing 50 mM Tris-HCl, pH 7.4; 150 mM NaCl, and 1 mM EDTA, in the presence of a protease inhibitor cocktail (1:100 dilution, Sigma-Aldrich, Saint Louis, MO, USA). The homogenate was then centrifuged at 10,000× *g* for 15 min at a temperature of 4 °C, and the supernatant was collected. The pellet was suspended in a lysis buffer supplemented with the protease inhibitor cocktail, and was centrifuged again. The supernatants were pooled and centrifuged at 15,000× *g* for 30 min at 4 °C. The final supernatants were stored at 4 °C for 1–2 h until use. To quantify the level of Aβ in the mice, we used a sandwich ELISA Kit (Innotest Amyloid-β_1–2_) from Innogenetics (Gent, Belgium), following the instructions of the manufacturer. The absorbance values were measured at 450 nm on a 96-well plate reader (NOVOstar OPTIMA, BMG Labtech, Offenburg, Germany).

### 4.8. WB

Protein samples, identically prepared as for ELISA, (20 μg/lane) were separated on a 15% SDS-polyacrylamide gel (80 V, 20 min at RT; than 130 V, 60 min at RT) or on a 4–12% NuPAGE^®^ Bis-Tris Gel (Thermo Fischer Scientific, Waltham, MA, USA, 200V, 30 min at RT) and transferred to a nitrocellulose membrane (Whatman, Maidstone, UK, 450 mA, 90 min at 4 °C). The membranes were washed twice with blocking solution (Tris-buffered saline (TBS) containing 0.1% Tween-20 and 5% bovine serum albumin (BSA)); then, they were incubated overnight at 4 °C with the primary antibodies (1:5000, rabbit Fe65 antibody, Thermo Fisher Scientific, Waltham, MA, USA; 1:1000, mouse 6E10 antibody, BioLegend, San Diego, CA, USA; 1:2000, rabbit pT668-APP antibody, GeneTex, Irvine, CA, USA; 1:20,000, mouse β-actin antibody, Sigma-Aldrich, St. Louis, MO, USA, 1:2000, rabbit AICD antibody, Sigma-Aldrich, St. Louis, MO, USA; 1:20,000, mouse PSD95 antibody, Invitrogen, Carlsbad, CA, USA; 1:50,000, mouse synaptophysin antibody, Invitrogen, Carlsbad, CA, USA; 1:200,000, rabbit GAPDH antibody, Cell Signaling, Leiden, Netherlands) diluted in the blocking solution. Subsequently, membranes were washed three times with washing buffer (TBS containing 0.1% Tween-20), and they were incubated with the corresponding secondary antibodies (1:5000, goat anti-rabbit or rabbit anti-mouse, Immuno Reagents, Raleigh, NC, USA) for 2 h at RT. The visualization was performed with the WesternBright™ ECL detection kit (Sycamore, Houston, TX, USA). Membranes were scanned with a Bio-Rad Molecular Imager^®^ ChemiDoc™ XRS+Imaging System (Bio-Rad Laboratories, Hercules, CA, USA) and evaluated with the Image Lab 4.0 software.

### 4.9. Quantification of Spine Density

Mice (three animals per group) were deeply anaesthetized using chloral-hydrate (1 mg∙kg^−1^). The brain was quickly removed from the skull and cut into blocks. For staining, an FD Rapid Golgi Stain TM Kit (FD Neuro Technologies, Consulting & Services, Inc., Columbia, MD, USA) was used as per the manufacturers’ instructions. All measurements were then conducted as described previously [54,55].

### 4.10. Immunohistochemistry

Mice (five animals per group) were anaesthetized with chloral-hydrate (1 mg∙kg^−1^) and perfused transcardially with PBS, followed by ice-cold 4% paraformaldehyde. The brains were removed, post-fixed in the same fixative, and immersed in a 30% sucrose solution containing 0.01% sodium-azide. They were sliced up from the dorsal to the ventral part of the HC, on a freezing microtome, into 20-μm-thick coronal sections (12 sections/animal/staining). In the free-floating sections, endogenous peroxidases were quenched with 0.3% H_2_O_2_ in PBS. The acquired sections were then subjected to further immunohistochemical experiments. For 4G8 staining, the sections were incubated in 100% formic acid for 1 min at RT to aid in the immunohistochemical detection of amyloid plaques. The sections were subsequently rinsed four times in PBS following the pretreatments. For GFAP stainings, the sections were blocked in a mixture of 8% normal goat serum (Sigma-Aldrich, Saint Louis, MO, USA), 0.3% BSA (Sigma-Aldrich, Saint Louis, MO, USA), and 0.3% Triton X-100 (Sigma-Aldrich, Saint Louis, MO, USA) in PBS for 1 h. For DCX and Iba1 labeling, sections were blocked in 0.1% BSA and 0.3% Triton X-100 in PBS for 1 h. Sections were incubated overnight at 4 °C with primary antibodies in the following dilutions: rabbit anti-Iba1 (1:3600; Code No.: 019-19741, Wako Chemicals GmbH, Neuss, Germany), mouse anti-GFAP (1:1500; Santa Cruz Biotechnology, Inc., Dallas, TX, USA), and anti-β-amyloid antibody (1:10,000; 4G8; BioLegend, San Diego, CA, USA). The next day, the sections were rinsed three times in PBS. For GFAP staining, the sections were treated with a polymer-based horseradish peroxidase (HRP)-amplifying system (Super Sensitive™ One-Step Polymer-HRP IHC Detection System, BioGenex, Fremont, CA, USA). The assay was used in accordance with the manufacturer’s instructions. For Iba1 and β-amyloid stainings, the sections were incubated with the corresponding secondary antibodies: biotinylated goat anti-rabbit (1:400; Jackson ImmunoResearch, West Grove, PA, USA) for 60 min and biotinylated goat anti-mouse (1:400; Thermo Fisher Scientific, Waltham, MA, USA) for 90 min, respectively. The sections were then rinsed three times in PBS and incubated with an avidin–biotin complex (VECTASTAIN^®^ Elite ABC-Peroxidase Kit; Vector Laboratories, Burlingame, CA, USA) for β-amyloid staining in 1:500, for 90 min, and for Iba1 staining in 1:400, for 60 min at RT. The peroxidase immunolabeling was developed for 30 min in 0.5 M Tris-HCl buffer (pH 7.7) with 3,3′-diaminobenzidine (DAB, 10 mM, Sigma-Aldrich, Saint Louis, MO, USA) at RT. Sections were mounted with dibutyl phthalate xylene (DPX, Sigma-Aldrich, Saint Louis, MO, USA) onto slides and cover-slipped.

### 4.11. Quantification of the Immunohistochemical Data 

Slides were scanned by a digital slide scanner (Mirax Midi, 3DHistech Ltd., Budapest, Hungary), equipped with a Pannoramic Viewer 1.15.4, CaseViewer 2.1 program and a QuantCenter, HistoQuant module (3DHistech Ltd., Budapest, Hungary). For quantification, all of the sections were analyzed. The HC and the CTX were manually outlined as the regions of interest (ROI). Antibody-positive cell types from the ROIs were counted and quantified. The percentile densities of microglia (Iba1+), astrocytes (GFAP+), and Aβ plaques were quantified by the quantification software. The cell density was represented as a percentage.

### 4.12. Statistical Analysis

Behavioral data were analyzed by mixed ANOVA, followed by Fisher’s LSD post hoc tests for multiple comparisons. The classical and modified ANOVA data evaluation methods applied for the raw measurements could detect only some significant effects without explaining their positions. Convenient post hoc analysis methods, like Student’s *t*-statistics, need a Gaussian distribution of the analysis data. Additionally, only the probability of the false positive decision error can be set up, and the probability of the false negative decision error remains unknown. Therefore, we developed and used nonparametric statistics. The one-response variable data (swimming times) were used to generate a two-variable model: (1) the reciprocal of the slope of the fitted straight line between the swimming time and the days (its negative values close to zero indicate an improvement in the memory and learning functions of the mice from day to day); (2) swimming time data of the last day (closeness to zero indicates an effective learning process). The two variables were combined, and evaluating them together gave more reliable multiple statistical results. For the correct estimation and use of the probability of the false positive decision error, a permutation test was applied, while, for the correct estimation and use of the probability of the false negative decision error, we used bootstrap resampling. In the case of both resampling methods, 50,000 resamples were generated independently, using an in-house developed Matlab script. The detailed explanations of the mathematical statistical development will be published elsewhere.

The results of spine density and immunohistochemistry experiments were calculated with one-way ANOVA followed by Fisher’s LSD post hoc tests. The WB and ELISA data did not follow normal (Gaussian) distribution; they were, therefore, analyzed with Kruskal–Wallis nonparametric tests, followed by Mann–Whitney U tests for multiple comparisons. Data were analyzed with SPSS (IBM SPSS Statistics 24), and were expressed as the mean ± standard error of the mean (SEM). Statistical significance was set at *p* ≤ 0.05.

## 5. Conclusions

In the present study, a novel compound is described, which was proven to significantly decrease the level of Aβ (soluble Aβ_1–42_ and amyloid plaques) in APP/PS1 mice. As a consequence, treatment with P33 reduced memory deficit and the activation of microglia and astrocytes. It also enhanced the dendritic spine densities in the transgenic animals. We hypothesize that the beneficial effects of P33 can be explained by its binding to the Fe65 protein, the overexpression of which is characteristic for AD patients, as well as for transgenic APP-overexpressing animal models. Therefore, the compound could serve as a starting point for the development of a drug family, which exerts its effect based presumably on a novel mechanism of action, focused on the modulation of the Fe65-dependent APP processing in a favorable manner.

## Abbreviations

RIP	regulated intramembrane proteolysis
APP	amyloid precursor protein
AICD	intracellular domain of APP
AD	Alzheimer’s disease
Aβ	beta amyloid
HEK293	human embryonic kidney cells 293
H4	human H4 neuroglioma cells
MDCK	Madin–Darby Canine Kidney cells
ITC	isothermal titration calorimetry
APP/PS1	APPswe/PS1ΔE9 double transgenic
WT	C57BL/6 wild type
MWM	Morris water maze
GFAP	glial fibrillary acidic protein
Iba1	ionized calcium-binding adapter molecule 1
DCC	*N*,*N*′-dicyclohexyl-carbodiimide
HOBt	1-hydroxybenzotriazole
HATU	1-[bis(dimethylamino)-methylene]-1H-1,2,3-triazolo[4,5-b]pyridinium-3-oxid hexafluorophos-phate
MBHA x HCl	4-methylbenz-hydryl-amine hydrochloride
DIPEA	*N*,*N*-diisopropylethylamine
TFA	trifluoroacetic acid
ACN	acetonitrile
RT	room temperature
HC	hippocampus
CTX	cerebral cortex
WB	Western blot
TBS	Tris-buffered saline
BSA	bovine serum albumin
DAB	3,3′-diaminobenzidine
DPX	dibutyl phthalate xylene
ROI	regions of interest
CNS	central nervous system
mFe65	mouse Fe65
hAPP	human APP
ELISA	enzyme-linked immunosorbent assay
PSD95	postsynaptic density protein 95
SYN	synaptophysin
NFT	neurofibrillary tangles
